# Functional RECAP (REpair CAPacity) assay identifies homologous recombination deficiency undetected by DNA-based BRCAness tests

**DOI:** 10.1038/s41388-022-02363-1

**Published:** 2022-06-03

**Authors:** Titia G. Meijer, Luan Nguyen, Arne Van Hoeck, Anieta M. Sieuwerts, Nicole S. Verkaik, Marjolijn M. Ladan, Kirsten Ruigrok-Ritstier, Carolien H. M. van Deurzen, Harmen J. G. van de Werken, Esther H. Lips, Sabine C. Linn, Yasin Memari, Helen Davies, Serena Nik-Zainal, Roland Kanaar, John W. M. Martens, Edwin Cuppen, Agnes Jager, Dik C. van Gent

**Affiliations:** 1 Department of Molecular Genetics, Erasmus MC Cancer Institute, Erasmus University Medical Center, Rotterdam, The Netherlands; 2 Department of Pathology, Erasmus MC Cancer Institute, Erasmus University Medical Center, Rotterdam, The Netherlands; 3 Oncode Institute, Utrecht, The Netherlands; 4 Department of Molecular Genetics, University Medical Center Utrecht, Utrecht, The Netherlands; 5 Department of Medical Oncology, Erasmus MC Cancer Institute, Erasmus University Medical Center, Rotterdam, The Netherlands; 6 Cancer Computational Biology Center, Erasmus MC Cancer Institute, Erasmus University Medical Center, Rotterdam, The Netherlands; 7 Department of Urology, Erasmus MC Cancer Institute, Erasmus University Medical Center, Rotterdam, The Netherlands; 8 Department of Immunology, Erasmus MC Cancer Institute, Erasmus University Medical Center, Rotterdam, The Netherlands; 9 Department of Molecular Pathology, The Netherlands Cancer Institute, Amsterdam, The Netherlands; 10 Department of Medical Oncology, The Netherlands Cancer Institute, Amsterdam, The Netherlands; 11 Department of Pathology, University Medical Centre Utrecht, Utrecht, The Netherlands; 12 Academic Department of Medical Genetics, Cambridge Biomedical Campus, University of Cambridge, Cambridge, UK; 13 MRC Cancer Unit, Cambridge Biomedical Campus, University of Cambridge, Cambridge, UK; 14 Science Park, Hartwig Medical Foundation, Amsterdam, The Netherlands

**Keywords:** Breast cancer, Homologous recombination

## Abstract

Germline *BRCA1/2* mutation status is predictive for response to Poly-[ADP-Ribose]-Polymerase (PARP) inhibitors in breast cancer (BC) patients. However, non-germline *BRCA1/2* mutated and homologous recombination repair deficient (HRD) tumors are likely also PARP-inhibitor sensitive. Clinical validity and utility of various HRD biomarkers are under investigation. The REpair CAPacity (RECAP) test is a functional method to select HRD tumors based on their inability to form RAD51 foci. We investigated whether this functional test defines a similar group of HRD tumors as DNA-based tests. An HRD enriched cohort (*n* = 71; 52 primary and 19 metastatic BCs) selected based on the RECAP test (26 RECAP-HRD; 37%), was subjected to DNA-based HRD tests (i.e., Classifier of HOmologous Recombination Deficiency (CHORD) and BRCA1/2-like classifier). Whole genome sequencing (WGS) was carried out for 38 primary and 19 metastatic BCs. The RECAP test identified all bi-allelic *BRCA* deficient samples (*n* = 15) in this cohort. RECAP status partially correlated with DNA-based HRD test outcomes (70% concordance for both RECAP-CHORD and RECAP-BRCA1/2-like classifier). RECAP selected additional samples unable to form RAD51 foci, suggesting that this functional assay identified deficiencies in other DNA repair genes, which could also result in PARP-inhibitor sensitivity. Direct comparison of these HRD tests in clinical trials will be required to evaluate the optimal predictive test for clinical decision making.

## Introduction

Optimal patient selection for Poly-[ADP-ribose]-polymerase (PARP) inhibitors and DNA double strand break (DSB) inducing chemotherapy is of great clinical importance. PARP-inhibitor treatment has proven effective for breast cancer (BC) patients with germline *BRCA1/2* mutations, with a focus shifting from treatment in the metastatic setting more towards adjuvant treatment in the primary setting [1–4]. Tumors with germline *BRCA1/2* mutations are often homologous recombination deficient (HRD) and therefore respond well to these inhibitors [5–7]. However, HRD also occurs in tumors without germline *BRCA1/2* mutations, indicating that the use of these therapies could be extended beyond germline *BRCA1/2* mutated cancers [8]. The underlying causes of HRD can be either BRCA-related (e.g., somatic *BRCA* mutations, *BRCA1* promoter methylation) or non-BRCA-related (e.g., mutations in other genes, such as *PALB2 and RAD51C* [9, 10]). Therefore, development of a robust HRD test to identify these HRD or BRCA-like tumors is of critical importance.

The MYRIAD/ MyChoice test, based on the levels of loss of heterozygosity (LOH), telomeric allelic imbalance (TAI) and large-scale transitions (LST) throughout the genome, is the only commercially available HRD test and therefore its analytic and clinical validity have been demonstrated in various studies [11–15]. However, a substantial group of patients who benefitted from PARP inhibitors or interstrand crosslinking agents (platinum salts) were not classified as HRD by this test [14, 15]. The BRCA1/2-like classifier is another type of HRD test, which used *BRCA1/2* mutated BCs to define a specific genomic pattern of copy-number variation, as detected by genome-wide SNP microarrays or low-pass sequencing [16, 17]. This test has shown promising analytical and clinical validity for predicting benefit of DNA DSB-inducing agents in retrospective studies [18–20] and prospective studies are ongoing to determine ultimate clinical utility (NCT02810743 and NCT02826512). More recently, HRD tests based on whole genome sequencing (WGS) data have been published, such as HRDetect and Classifier of HOmologous Recombination Deficiency (CHORD) [10, 21]. These algorithms analyze genome-wide mutational scars that result from imprecise DNA repair processes correlated with HRD in *BRCA* gene mutated cancers. Although these studies show clear analytical validity, thorough clinical validation is still required for WGS-based HRD tests [22, 23].

All HRD tests mentioned above are DNA-based, scoring mutagenic events that accumulated over the course of tumor evolution. However, selection pressure due to systemic treatment(s) may induce resistance mechanisms, especially in the metastatic setting, which is expected to hamper faithful HRD detection. Therefore, functional HR assessment just before the start of treatment would have several advantages. The REpair CAPacity (RECAP) test determines the HR phenotype by measuring RAD51 foci formation in proliferating cells (in the S or G2 phase of the cell cycle) after ex vivo irradiation of fresh BC tissue, enabling determination of the HRD status functionally [24–26]. Its clinical validity is currently being investigated in a small proof of concept study, the FUTURE trial (NL8099).

Here, we investigated whether a functional test defines a similar group of HRD tumors as DNA-based tests for primary and metastatic BC lesions. More specifically, the HRDetect, CHORD and BRCA1/2-like classifier algorithms were performed on tumors with known functional HRD status, as determined by RECAP.

## Results

### BRCA status of an HRD enriched cohort of primary and metastatic breast cancers

**Fig. 1:**
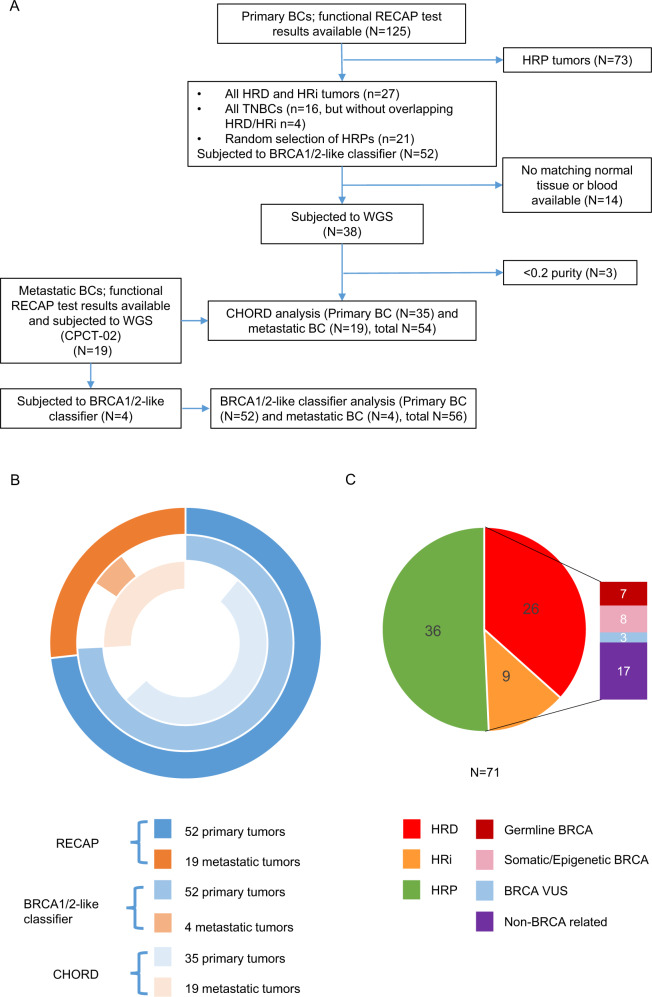
Selection of an HRD enriched cohort. **A** Flowchart illustrating the inclusion of tumors. **B** Graphic illustration of selection of tumors for different HRD tests. **C** Numbers of tumors that were found HRP/HRi/HRD according to the RECAP test. The cause for HRD/HRi is depicted. Only bi-allelic inactivation through germline and somatic mutations in combination with LOH are depicted in this graph.

Previously, the RECAP test was performed on primary and metastatic BCs [24–26]. Here, a cohort (*n* = 71; 52 primary and 19 metastatic lesions) enriched for HRD tumors was selected based on the RECAP test, consisting of 26 RECAP-HRD (37%), 9 RECAP homologous recombination intermediate-(HRi) (13%) and 36 RECAP homologous recombination proficient -(HRP) (51%) tumors (Fig. 1 and Table S1).

Eight tumors (11%) were derived from patients with germline *BRCA1/2* mutations, two tumors (3%) harbored somatic *BRCA1/2* mutations and six tumors (8%) showed *BRCA1* promoter hypermethylation (Table S1). *BRCA* inactivation was bi-allelic in 15 of these 16 tumors. Tumor M273 harbored a germline *BRCA2* mutation (c.3847_3848delGT p.Val1283fs) without loss of the other allele (mono-allelic). Among the nine tumors with germline *BRCA1/2* mutations, one tumor (M248) harbored a germline *BRCA1* variant (c.5309G>T p.Gly1770Val) that was not classified as pathogenic in CLINVAR. However, the variant was classified as pathogenic based on a recent report [27], and therefore regarded as pathogenic in our analysis. The nonsense substitution mutation in *BRCA2* (c.7285G>T, p.Glu2429X) in tumor M096 has not been described before and is thus not classified in CLINVAR. However, based on its predicted effect, we classified this variant as pathogenic in our analysis.

### RECAP identifies all bi-allelic BRCA deficient tumors

The RECAP test identified all 15 bi-allelic *BRCA* deficient samples in this cohort as either HRD or HRi (15/35 = 43%) (Fig. S3 and Table S1). In 3 RECAP-HRD/HRi tumors a variant of unknown significance (VUS) was detected in *BRCA1/2* in combination with LOH (3/35 = 9%). These tumors were not regarded as non-BRCA HRD/HRi or BRCA-related HRD/HRi, as their BRCA status is uncertain. The remaining 17 RECAP-HRD/HRi tumors did not show bi-allelic *BRCA* inactivation through a pathogenic mutation or promoter hypermethylation, and were therefore regarded as non-BRCA HRD/HRi (17/35 = 49%) (Fig. 1). We did not detect pathogenic mutations in other HR genes that could explain the HRD status of the non-BRCA HRD/HRi samples in this cohort, such as *PALB2* and *RAD51C*.

In the RECAP-HRP tumors no bi-allelic *BRCA1/2* deficiencies were identified. In three RECAP-HRP tumors a VUS was detected in *BRCA1/2*, in two cases without LOH and one in combination with LOH (3/36 = 8%). One RECAP-HRP tumor (M273) harbored a germline *BRCA2* mutation (c.3847_3848delGT p.Val1283fs) without loss of the other allele (mono-allelic).

### RECAP status compared to BRCA1/2-like classifier and DNA-based BRCAness outcomes

Since there is not yet a golden standard for measuring HRD, we investigated whether the RECAP-HRD/HRi tumors would also be detected by other HRD tests—the WGS-based HRD prediction algorithms CHORD and HRDetect and the BRCA1/2-like classifier (Fig. S4, Table S2).

**Fig. 2:**
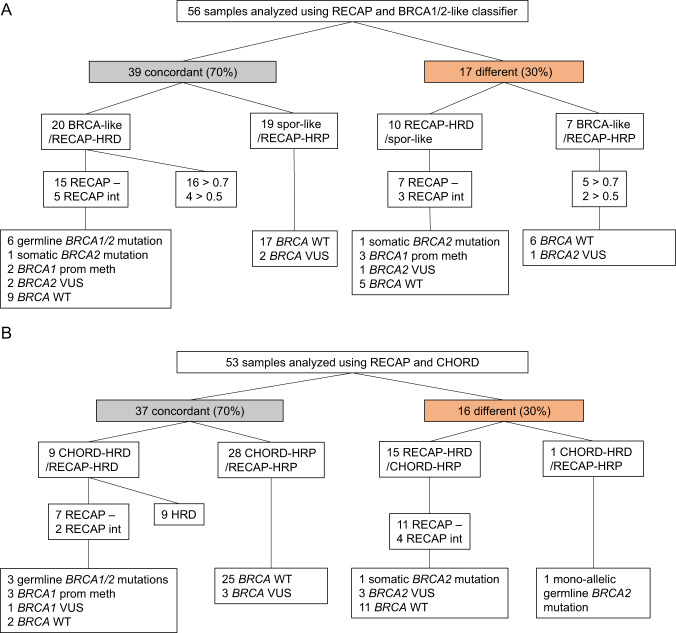
Comparisons of RECAP test, BRCA1/2-like classifier and CHORD test. **A** Comparison of RECAP test and BRCA1/2-like classifier. According to the RECAP test, HRD is described as tumors showing RAD51 foci in <20% of tumor cells in S-phase. HRi tumors show RAD51 foci in more than 20%, but less than 50%, of tumor cells in S-phase. According to the BRCA1/2-like classifier, tumors with scores of >0.7 are HRD and between 0.5 and 0.7 are HRi. Spor-like = HRP according to BRCA1/2-like classifier. *BRCA* defects led to bi-allelic inactivation unless otherwise specified. **B** Comparison of RECAP test and CHORD algorithm. According to CHORD, tumors with scores of >0.7 are HRD, scores of <0.7 are HRP. *BRCA* defects led to bi-allelic inactivation unless otherwise specified.

We analyzed 52 primary tumors and four metastases by both RECAP and BRCA1/2-like classifier (*n* = 56) (Fig. 2A). The BRCA1/2-like classifier found 22 HRD (39%), five HRi (9%) and 29 HRP (52%) tumors (Fig. S4). The BRCA1/2-like classifier identified nine out of 13 (69%) bi-allelic *BRCA* deficient samples which were subjected to the BRCA1/2-like classifier as either HRD or HRi (Fig. S4). In three HRD/HRi tumors a VUS was detected in *BRCA1/2* in combination with LOH. The remaining 15 HRD/HRi tumors did not show bi-allelic *BRCA* inactivation (non-BRCA HRD/HRi). Compared to RECAP, the BRCA1/2-like classifier identified seven extra HRD/HRi tumors, among which no bi-allelic *BRCA* inactivation was found. Compared to the BRCA1/2-like classifier, RECAP identified ten extra tumors as HRD, among which were four bi-allelic *BRCA* deficient tumors: one tumor (M096) with a somatic *BRCA1/2* mutation, three tumors (M119, M141, and M277) with *BRCA1* promoter methylation, as well as one tumor with a *BRCA2* VUS and five *BRCA* wild-type tumors. RECAP and BRCA1/2-like classifier classified 30% (17/56) of the tumors differentially (Fig. 2A).

The comparison between RECAP and WGS-based DNA tests was first made for the primary tumors with WGS data available (*n* = 35). Two tumors (M096 and M133) did not meet the quality control standards of the HRDetect test. The normal control of tumor sample M096 showed significant contamination with tumor DNA. We found a perfect correlation between the DNA-based BRCAness tests CHORD and HRDetect for the other primary BC samples (*n* = 33) (Fig. S5), therefore only the CHORD results were used in the comparisons among HRD tests. However, a comparison with HRDetect would result in the exact same numbers. The metastatic samples were also analyzed by a DNA-based BRCAness test (CHORD). The CHORD algorithm was applied to 35 primary tumors and 18 metastases (*n* = 53). The CHORD algorithm found 10 HRD (19%) and 43 HRP (81%) tumors (Fig. S4). CHORD identified seven out of eight (88%) bi-allelic *BRCA* deficient samples which were subjected to CHORD as HRD. Next, the CHORD algorithm identified one tumor (M273) with mono-allelic *BRCA2* inactivation and two tumors that did not show bi-allelic *BRCA* inactivation as HRD (non-BRCA HRD) (Fig. S3). The HRD tumor with mono-allelic pathogenic germline *BRCA2* mutation (M273) was not identified by the other tests. Fifteen tumors were HRD according to the RECAP test, but not according to CHORD. Among these tumors was one bi-allelic *BRCA* deficient tumor with a somatic *BRCA1/2* mutation (M096)), three harbored a *BRCA* VUS and eleven tumors were *BRCA* wild-type. CHORD and RECAP classified 30% (16/53) of the tumors differentially (Fig. 2B).

CHORD and BRCA1/2-like classifier (*n* = 38), performed on 35 primary tumors and three metastases, showed discrepant results in 34% (13/38) of the samples (Fig. S6). One tumor, with *BRCA1* promoter methylation, was HRD according to CHORD but not to the BRCA1/2-like classifier. The BRCA1/2-like classifier identified 12 extra tumors as HRD compared to CHORD. Three of these tumors harbored VUSes and nine were *BRCA* wild-type.

**Fig. 3:**
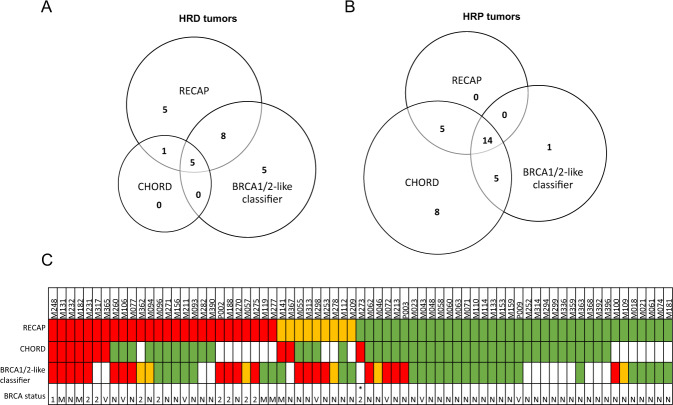
HRD status of 71 breast cancers as defined by different HRD tests. **A**, **B** Venn-diagrams showing overlap among RECAP, BRCA1/2-like classifier and CHORD test outcomes and comparisons of RECAP test and BRCA1/2-like classifier and CHORD test. **A** HRD/HRi tumors and (**B**) HRP tumors as identified by one or multiple HRD tests. The numbers in the circles correspond to the number of HRD/HRi tumors (**A**) or HRP tumors (**B**) identified by that test. **C** HRD status of BRCA inactivated cases per HRD test. Green = HRP, orange = HRi, red = HRD. 1 = *BRCA1* mutation, 2 = *BRCA2* mutation, M = *BRCA1* methylation, V = *BRCA1/2* VUS, N = normal BRCA status. *mono-allelic *BRCA2* inactivation.

**Fig. 4:**
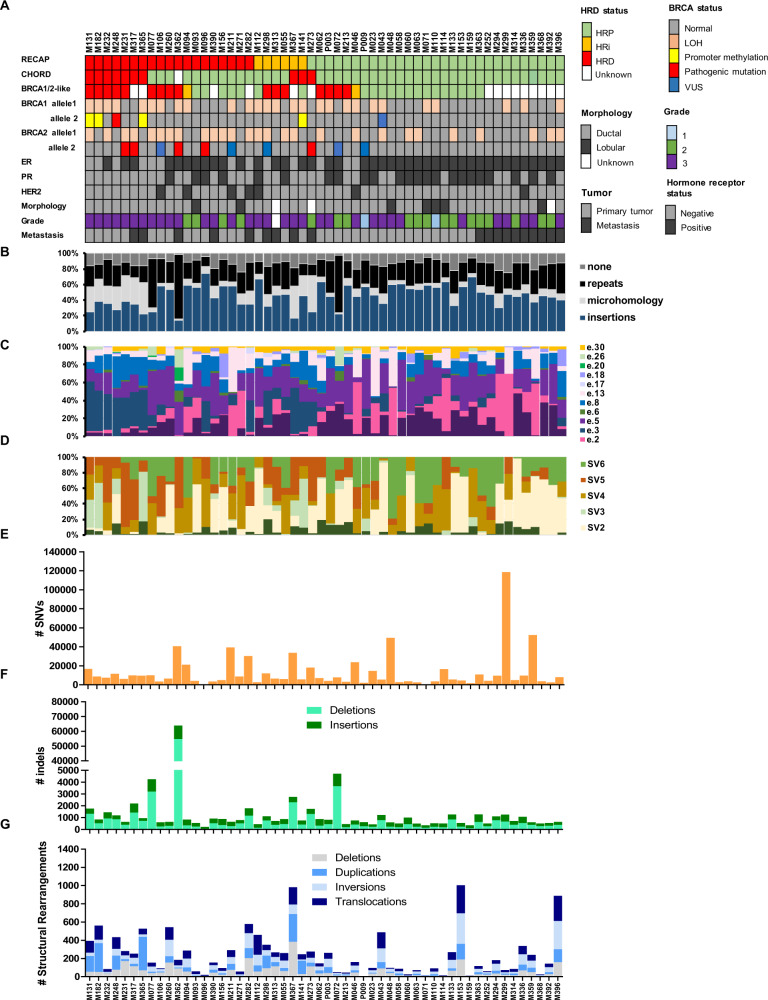
Somatic mutational landscape of 54 breast cancers enriched for HRD. The 54 tumors that were whole genome sequenced are depicted in this figure. **A** Clinical and histological parameters for each tumor: HRD status per test, bi-allelic *BRCA* status (≥class 2 VUSes are included in this figure), estrogen receptor (ER), progesterone receptor (PR), human epidermal growth factor receptor 2 (HER2), tumor morphology (ductal or lobular), histological grade and whether the tumor was a primary or metastatic tumor. **B** The relative contributions of different types of indels. Insertions: all insertions. Repeats: indels in repeat regions were defined as the presence of ≥1 copy of the indel sequence downstream (i.e., in the 3′ direction) from the breakpoint, where sequence length must be <50 basepairs. Microhomology: indels with flanking microhomology were defined as the presence of the following sequence features up or downstream from the breakpoint: (i) ≥1 copy of the indel sequence if the indel sequence length is ≥50 bp; (ii) ≥2 bp sequence identity to the indel sequence; or (iii) ≥1 bp sequence identity if the indel sequence length is ≥3 bp. For (ii) and (iii) the number of up or downstream bases searched was equal to the length of the indel. None: other deletions [10]. **C** The relative contributions of twelve substitution signatures [28]. **D** The relative contributions of six rearrangement signatures [28]. **E** Number of somatic single nucleotide variants (SNVs). **F** Number of somatic insertions and deletions (indels). **G** Number and type of somatic structural rearrangements.

Overall, in 38 cases all three HRD tests were successfully performed. Five tumors were identified as HRD (Fig. 3A) and 14 tumors as HRP by all three tests (Fig. 3B). Among the five tumors that were found HRD by all three tests four tumors showed bi-allelic *BRCA1/2* inactivation (germline mutations (*n* = 2) or promoter methylation (*n* = 2)) (Fig. 4). The other tumor (M232) only showed LOH at the *BRCA1* and *BRCA2* loci without loss of function in the other allele (Fig. 4A). Among the RECAP-HRD/HRi tumors in this cohort, 24 out of all 35 (69%) RECAP-HRD/HRi and 10 out of the 17 (59%) non-BRCA RECAP-HRD/HRi tumors were also classified as HRD/HRi by at least one other HRD test (Table S1, Figs. 3C, S3).

### Concordance of HRD test results among primary and metastatic tumors

Among the 19 metastatic lesions were 8 (42%) RECAP-HRD/HRi tumors and among the 52 primary tumors were 27 (52%) RECAP-HRD/HRi tumors. RECAP and BRCA1/2-like classifier classified 17 samples differentially among the 52 primary tumors (33%; 17/52) and showed concordance among the metastatic lesions (0%; 0/4) (Table S1). CHORD and RECAP classified 11 samples differentially among the 35 primary tumors (31%; 11/35) and 5 samples among the 18 metastases (28%; 5/18) (Table S1). CHORD and BRCA1/2-like classifier classified 11 samples differentially among the 35 primary tumors (31%; 11/35) and 2 samples among the 3 metastases (67%; 2/3) (Table S1).

### Somatic landscape of BRCA and non-BRCA RECAP-HRD tumors

WGS data was used to search for features other than *BRCA1/2* inactivation that characterize functional HRD tumors. RECAP-HRD tumors harbored more deletions (indels), and specifically deletions with microhomology, which is the main feature for WGS-based tools like HRDetect and CHORD and can be a result of an alternative error-prone repair mechanism for DSBs (microhomology-mediated end joining) (Fig. S7). Also, RECAP-HRD tumors harbored more large rearrangements; more specifically structural deletions and translocations (Fig. S7). We determined the contribution of the twelve substitution and six rearrangement signatures described previously for BC [28] (Figs. 4, S8, S9). RECAP-HRD tumors had a higher burden of substitution signature 3 than HRP tumors, although up to half of the HRD tumors had a signature 3 contribution similar to HRP tumors (Fig. S8).

In this cohort, fifty-seven out of the 93 previously identified BC driver genes [28] contained a mono-allelic somatic mutation, deep deletion or high gain in at least one tumor (Fig. S10). The four most often affected genes were *TP53* (19 cases), *CCND1* (10 cases), *PTEN* (9 cases) and *ZNF703* (9 cases). *TP53* mutations were present in 54% (13/24) of RECAP-HRD/HRi tumors versus 23% (6/26) of RECAP-HRP tumors. *PTEN* mutations were present in both RECAP-HRD/HRi and RECAP-HRP tumors, 17% (4/24) and 19% (5/26), respectively. Two genes differentiated between HRD and HRP tumors; *ARID1A* was only affected in RECAP-HRD tumors (n = 4), whereas *PIK3CA* was only affected in HRP tumors (*n* = 4) (Fig. S10). RECAP-HRD tumors M362, M231 and M077 were found to be MSI, due to a large deletion in *MLH1* or *PMS2* or *MLH1* promoter hypermethylation [24], respectively. We did not find bi-allelic inactivation of HR genes that could explain the HRD status of the non-BRCA tumors, such as *PALB2*, *BRIP1*, *BARD1*, *RAD51C* [29].

Non-BRCA RECAP-HRD tumors did not show significantly higher average contributions of signatures related to *BRCA* (Fig. S11). The mutational landscape of non-BRCA HRD tumors was heterogeneous: while some tumors had high numbers of SNVs and presence of APOBEC signatures (2 and 13) (M094 and M271) and others showed mutational signatures associated with *BRCA* defects (M232, M282 and M367) or MSI (M077), most had quiet genomes with only a few somatic alterations (M093, M390, M156, M112, M313, M055, M260) (Fig. 4) [24].

## Discussion

Here, we investigated whether the functional RECAP test defines a similar group of HRD tumors as DNA-based tests. RECAP-HRD tumors were frequently not identified as HRD by (one of the) other tests, whereas some RECAP-HRP tumors were assigned to the HRD group by other tests. This led to a relatively high level of discrepancy (approximately 30% discordance among tests). Thus, each one of the different methods to define ‘HRD’ should be evaluated carefully and clinical validation is required before they can be used in a clinical setting.

In this study, we assessed analytical validity, which is necessary before clinical validity and utility can be investigated. Analytical validity encompasses many aspects, such as feasibility, reproducibility and robustness. We focus on accuracy of HR measurement, since feasibility and reproducibility have been described for each test individually [16, 17, 21, 24]. Without a golden standard HRD test, only the differences among the HRD tests in identifying bi-allelic *BRCA1/2* deficient tumors and putative HRD tumors without *BRCA1/2* defects can be described. The RECAP test identified all bi-allelic *BRCA* deficient samples in this cohort as either HRD or HRi. The BRCA1/2-like classifier identified seven and CHORD one extra HRD/HRi tumors compared to RECAP, among which no bi-allelic *BRCA* inactivation was found. The BRCA1/2-like classifier missed four and CHORD one bi-allelic *BRCA* deficient tumor(s). RECAP identified 11 extra HRD/HRi tumors compared to the BRCA1/2-like classifier and CHORD.

Before elaborating on the possible biological explanations for these discrepancies, technical reasons should be considered. For the tumor with a somatic *BRCA2* mutation with LOH (M096), which was not identified as HRD by the BRCA1/2-like classifier and CHORD, the normal control was shown to contain significant contamination with tumor DNA, which is likely to have compromised the somatic mutation calling. Although this may have influenced CHORD results, this cannot explain the HRP score for the BRCA1/2-like classifier, which only requires tumor tissue.

One of the discrepant tumors in the current cohort (M273), was a metastatic tumor with a germline *BRCA2* mutation without inactivity of the other allele. Since this tumor also carried a germline *CHEK2* mutation (c.1100delC; p.Thr367fs) with LOH, it is more likely that this *CHEK2* mutation has driven tumorigenesis [26]. However, it is possible that reversion of an undetected second mutation in *BRCA2* has occurred in the metastasis and therefore transient loss of *BRCA2* expression in the past could explain the fact that this tumor was found HRD (BRCA2-type) by CHORD. This example highlights the advantage of functional HR assessment in the metastatic setting.

Various possible reasons for the discrepancies between RECAP and DNA-based tests exist. First, the differences could be explained by their specific working mechanisms and the actual substrate measured. RECAP directly measures whether the HR pathway functions properly by analyzing RAD51 foci formation after irradiation. Reversion of the HR status due to previous systemic treatment(s) will therefore directly change the functional RECAP outcome. DNA-based tests measure alterations in the tumor genome that result from imprecise DNA repair processes correlated with HRD in *BRCA* mutated cancers. Thus, DNA-based tests score historic events and may be unreliable for detection of HR status after previous (DSB-inducing) treatments. This suggests that DNA-based HRD tests are especially suitable at primary diagnosis, before selection pressure and resistance mechanisms have occurred due to systemic treatment(s). Nevertheless, our analysis shows that discordance occurred among both primary and metastatic tumors (Fig. 4). Therefore, the discordance among the tests is not solely due to HR reversion.

Second, one of the main features for WGS-based tools like HRDetect and CHORD is presence of deletions with microhomology, which can be a result of an alternative error-prone repair mechanism for DSBs (microhomology-mediated end joining). Approximately half of the RECAP-HRD/HRi tumors do not harbor many deletions with microhomology (Fig. S7), which may explain the discordance between CHORD and RECAP. Moreover, substitution signature 3 (enriched in C>G substitutions) has previously been found to correlate with the presence of inactivating *BRCA1* and *BRCA2* mutations [21, 30]. Similarly, substitution signature 3 could not be used to discriminate RECAP HRD and HRP tumors (Fig. S8). These discrepancies could either mean that HRD caused by defects in other HR genes do not (always) result in increased levels of microhomology-mediated end-joining and signature 3 mutations or the discrepant cases could be incorrectly assigned to the HRD group by RECAP. It is possible that increased use of microhomologies at junctions is not a general feature of defective HR repair, because it is a specific adaptation to *BRCA* gene defects, but not to (all) other HR defects. Direct evaluation of PARPi sensitivity will be required to settle this issue.

Third, the discrepancies could result from a proportion of the *BRCA*-related tumors harboring somatic or *BRCA1* promoter methylation, rather than germline *BRCA* mutations. The specific alterations could have happened relatively late in the tumor evolution and methylation can be unstable, leading to low numbers of *BRCA*-related mutations and structural changes which are below the threshold of detection of DNA-based HRD tests. This explanation could be valid for the three tumors with *BRCA1* promoter methylation and the tumor with a somatic *BRCA2* mutation with LOH, which were not identified as HRD by BRCA1/2-like classifier and both BRCA1/2-like classifier and CHORD, respectively.

A limitation to the current study is enrichment for RECAP-HRD samples, therefore this study does not reflect the natural occurrence of HRD in unselected BCs. Also, the number of metastatic tumors included is relatively low. Furthermore, only analytical validity and not clinical validity and utility are assessed here, since data on the predictive value for therapy response is not yet available for RECAP and CHORD.

The HRD tests applied in this study have their own advantages and disadvantages, which are discussed in more detail elsewhere [31]. In short, the main advantage of the RECAP test is its functional character, whereas DNA-based HRD tests score mutagenic events that accumulated over the course of tumor evolution. Regarding input material, RECAP requires a fresh biopsy and CHORD requires a fresh frozen biopsy, while for the BRCA1/2-like classifier FFPE material is sufficient. To overcome the need for a fresh biopsy, others have used RAD51 foci as a potential biomarker without prior induction of DNA damage. The RAD51 test on Formalin-Fixed Paraffin-Embedded (FFPE) material has shown predictive value in the randomized GeparSixto trial and in the non-randomized PETREMAC trial. Therefore, presence of endogenous DNA damage has been shown in a substantial fraction of untreated Triple Negative Breast Cancers (TNBCs) [32, 33]. One of the limitations of the RECAP test is that it uses absence of RAD51 foci as a read-out, so technically failed assays may result in RECAP HRD scores for HRP tumors, while the other tests detect presence of a genomic alteration. To minimize the chance of false HRD scores, repeated immunofluorescent stainings are performed and stromal cells with RAD51 foci are considered internal controls. A limitation of the BRCA1/2-like classifier is that the test is less reliable on tumor tissue with admixture of many normal cells (i.e., tumor cell percentage <50%) [34]. Therefore, patients who have a *BRCA1* promotor hypermethylation, regardless of the BRCA1/2-like classifier outcome, are also eligible for two ongoing prospective studies where the BRCA1/2 classifier is used (NCT02810743 and NCT02826512). CHORD has the drawback that presence of MSI hinders adequate HRD prediction (as exemplified here in tumor M362). However, WGS-based methods like CHORD and HRDetect are not only accurate for detecting damage to the DNA, but have the advantage that other potentially relevant biomarkers (e.g., HER2 amplification, microsatellite instability, oncogene/tumor suppressor mutations, etc) as well as mutational status of all driver and resistance HR variants are simultaneously detected.

Several HRD tumors harbored *BRCA1/2* VUSes and showed LOH, suggesting that this could be the cause for HRD. In one case (M298), the variant (c.9104A>C, p.Tyr3035Ser) in *BRCA2* has been reported previously to reduce BRCA2 protein activity and increase BC risk, however conflicting interpretations of its pathogenicity exist [35]. For the remaining variants, the likelihood of the VUS causing HRD increases when the tumor that harbors the VUS is found HRD by more than one HRD test. Three additional HRD tumors harbored VUSes, of which tumor M106 was found HRD by two tests (RECAP and BRCA1/2-like classifier) and M211 and M072 by one test only (RECAP, BRCA1/2-like classifier, respectively). For clinical practice, it is unclear whether patients harboring a *BRCA* VUS would benefit from DSB-inducing therapies or PARP-inhibitor treatment. Further investigation is needed to determine whether HRD testing could aid in predicting pathogenicity of VUSes and thereby predicting therapy response.

In conclusion, different HRD tests only partially identify the same population of BC patients. Therefore, predictive value should be determined for each individual test in order to select the test with the highest clinical utility. Also, the HRD test with the highest clinical utility can differ in the primary or metastatic setting. Clinical trials have been initiated in which patients with metastatic BC are subjected to the RECAP test or both the RECAP test and BRCA1/2-like classifier before receiving PARP-inhibitor treatment (respectively: NL8099 and NCT02810743). Preferably, multiple HRD tests should be included in a controlled clinical trial to determine how well these tests predict meaningful clinical benefit from PARP inhibitors, as it is not yet possible to choose one ultimate HRD test.

## Methods

### Breast cancer specimen collection

Residual fresh BC tissue was collected from resection specimens of the breast in the Erasmus MC Cancer Institute, Haven hospital and Maasstad hospital in Rotterdam, The Netherlands, according to the “Code of proper secondary use of human tissue in the Netherlands” established by the Dutch Federation of Medical Scientific Societies and approved by the local Medical Ethical committees (MEC-11-098) [24, 25]. Biopsies from metastatic BC lesions were performed as previously described [26], after registration and written informed consent. The study (NL49306.078.14/MEC14-295) was approved by the medical ethics committee of the Erasmus MC. Objective clinical response to relevant therapies was determined according to RECIST 1.1 criteria [36].

### Breast cancers with known functional HR status

The functional HRD status of a cohort of primary BCs (*n* = 125) was previously determined by RECAP testing [24]. In brief, presence of RAD51 foci was determined in S/G2 cells only, which stain positive for Geminin. At least 30 Geminin expressing cells were counted per tumor sample. A cell was considered RAD51 positive when at least 5 RAD51 foci could be detected (Fig. S1). We used an additional double strand DNA break (DSB) marker (yH2AX) to determine that the irradiation was successful and DSBs were present in all samples. Tumors were classified as HRP, HRD or HRi when more than 50%, less than 20% or between 20–50% of geminin positive cells showed ≥5 RAD51 foci, respectively. A selection (*n* = 52) of these tumors was taken for further genomic analyses and comparison to other HRD tests (Fig. 1). This selection contained all HRD and HRi tumors from the initial set, regardless of Estrogen Receptor and Progesteron Receptor (ER/PR) status, as well as all TNBCs regardless of HR status. Next, 21 tumors that were HRP as well as ER/PR+ (defined as ≥10% protein expression) were selected based on tissue availability. Matching normal tissue or blood was available for 38 of these tumors, which were subsequently subjected to WGS. Three samples were excluded for further analysis due to a low purity score (<0.2), thus WGS data was present for 35 primary BCs. Additionally, 19 metastatic BCs with known RECAP status, for which WGS data were available, were also included. The BRCA1/2-like classifier was performed on 52 primary and 4 metastatic BCs.

### DNA isolation

DNA was isolated from 30 µm sections of fresh frozen primary tumor and adjacent normal tissue and quality checks of isolated DNA were performed as described previously [24]. DNA isolation from the metastatic biopsies and matching normal blood samples was performed as described previously [37].

### BRCA1/2-like classifier

The BRCA1/2-like classifier was performed by either Nimblegen arrays or low coverage copy-number sequencing and has been described previously [38, 39]. The cutoff was set at 0.7 for classification of a tumor as BRCA1-like or BRCA2 like, an intermediate score was defined as higher than 0.5 but lower than 0.7. The BRCA1-like and BRCA2-like classifier tool is available via http://ccb.nki.nl/software/nkiBRCA/.

### Whole genome sequencing

DNA libraries for Illumina sequencing were generated using standard protocols (Illumina, San Diego, CA, USA) and subsequently sequenced in an Illumina X-Ten sequencer by Novogene (Cambridge, UK). Matched tumor and germline DNA underwent whole genome sequencing to an average depth base coverage of 60× (range 39.9–97.9) and 25x (range 20.6–36.1) (Fig. S2), respectively. WGS of the tumor DNA from the metastatic biopsies and germline DNA from blood was performed as described previously [37]. Sequencing alignment and variant calling were performed as described [40], with a change in the structural variant calling procedure. Briefly, sequence reads were mapped against human reference genome GRCh37 using Burrows–Wheeler Aligner (v0.7.5a) with duplicates marked for filtering. Somatic variant calling was performed by Strelka (v1.0.14) with optimized settings and post-calling filtering, and indels were realigned using GATK (v3.4.46) IndelRealigner. Structural variants were called using GRIDSS (v1.8.0) and copy-number calling was performed using PURPLE (PURity and PLoidy Estimator) [41]. Bi-allelic mutation status of HR related genes was performed using an in-house pipeline that interprets copy-number, and germline and somatic SNV/indel data from the HMF variant calling pipeline to determine bi-allelic gene status (https://github.com/UMCUGenetics/hmfGeneAnnotation). WGS data is publicly available via the European Genome-Phenome archive (study ID: EGAS00001005572, dataset ID: EGAD00001008027). Non-*BRCA* HRD/HRi tumors were defined as tumors that were identified as RECAP-HRD/HRi and did not show bi-allelic *BRCA* inactivation through a pathogenic mutation or promoter hypermethylation.

### Whole genome sequencing based HRD prediction

CHORD was used for HRD predictions [10]. CHORD is a random forest model that calculates the probability of HRD based on a training set with known BRCA1 and BRCA2-deficient and -proficient tumors and uses the relative contributions of specific mutation contexts within a sample, with the most important features being deletions with flanking microhomology and 1–10 kb duplications. CHORD discriminates between BRCA1 and BRCA2-type HRD. Samples with a probability of HRD ≥ 0.5 were considered HRD. The mutation contexts used as input for CHORD were extracted from somatic variant data using mutSigExtractor [41]. CHORD scores cannot be determined on microsatellite unstable (MSI) tumors (>14.000 repetitive indels).

CHORD HRD predictions were compared with HRDetect HRD predictions in the 35 primary breast tumors. HRDetect was performed as described previously [21]. Due to failed quality control in 2 samples, HRDetect scores were available for 33 primary breast tumors.

### BRCA1 promoter methylation

*BRCA1* promoter methylation was analyzed by MLPA as described previously [24].

### Statistics

Statistical analyses were all two sided and performed using Graphpad Prism v6.0 (San Diego, CA, USA). Significance was calculated by Mann–Whitney test for continuous data and by Wilcoxon signed rank test for relative proportions of signatures. *P* values of <0.05 were considered significant.

## Supplementary information

Supplementary table 1

Supplementary table 2

Supplementary figures

Supplementary figure legends

## Data Availability

The WGS data have been deposited in the European Genome-phenome Archive (EGA) repository under the accession code (study ID: EGAS00001005572, dataset ID: EGAD00001008027).
