# Supplementary material for: Functional RECAP (REpair CAPacity) assay identifies homologous recombination deficiency undetected by DNA-based BRCAness tests
Source: Oncogene. 2022 Jun 3;41(26):3498–506. doi: 10.1038/s41388-022-02363-1 (PMC9232391; doi:10.1038/s41388-022-02363-1)
Supplement: Supplementary file 3 — Supplementary figures [file 41388_2022_2363_MOESM3_ESM.pdf]

Figure S1

RAD51 immunofluorescence

BRCA carrier:
RAD51 negative/ HRD

Control patient:
RAD51 positive/HR+

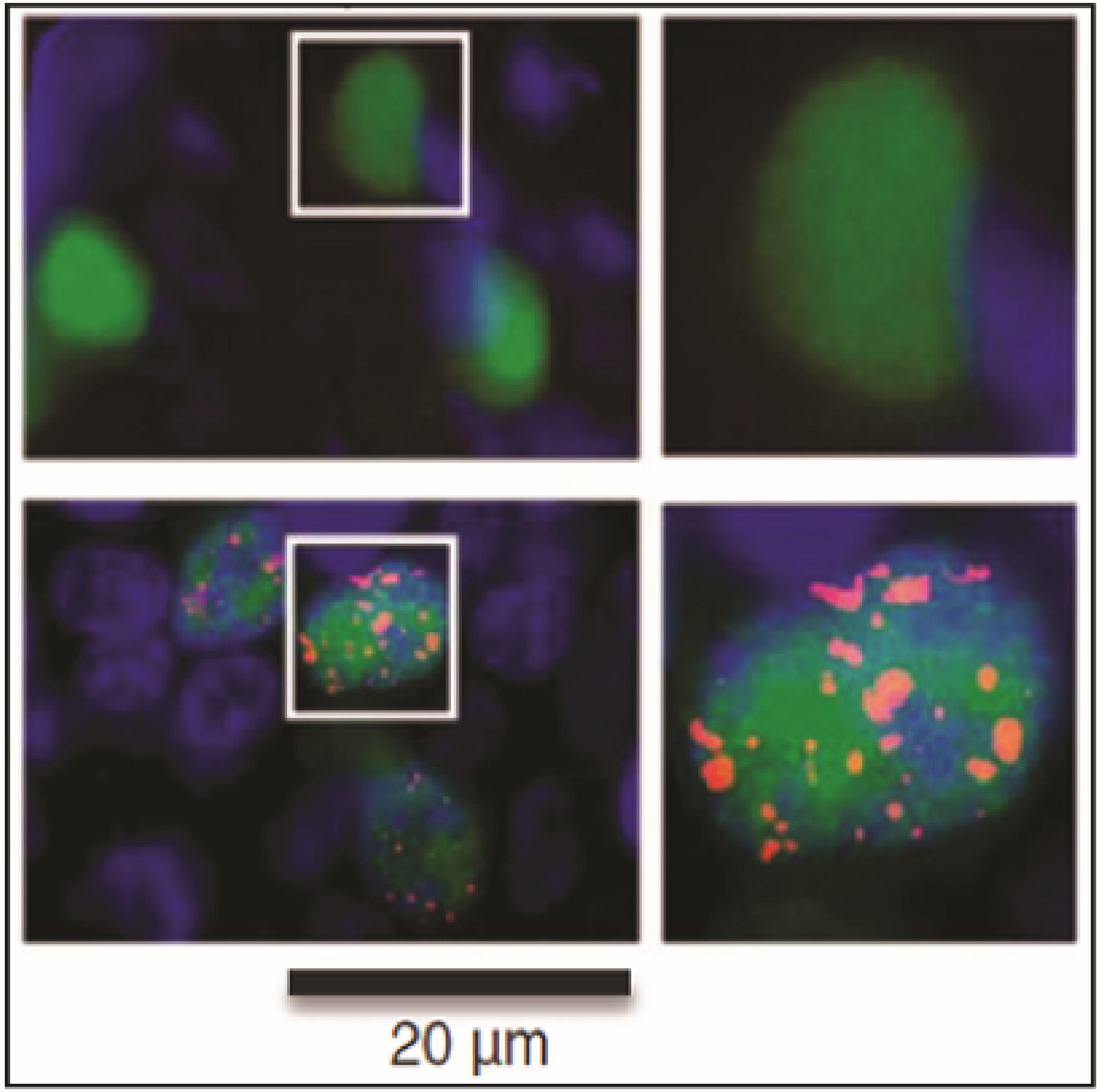

Figure S2

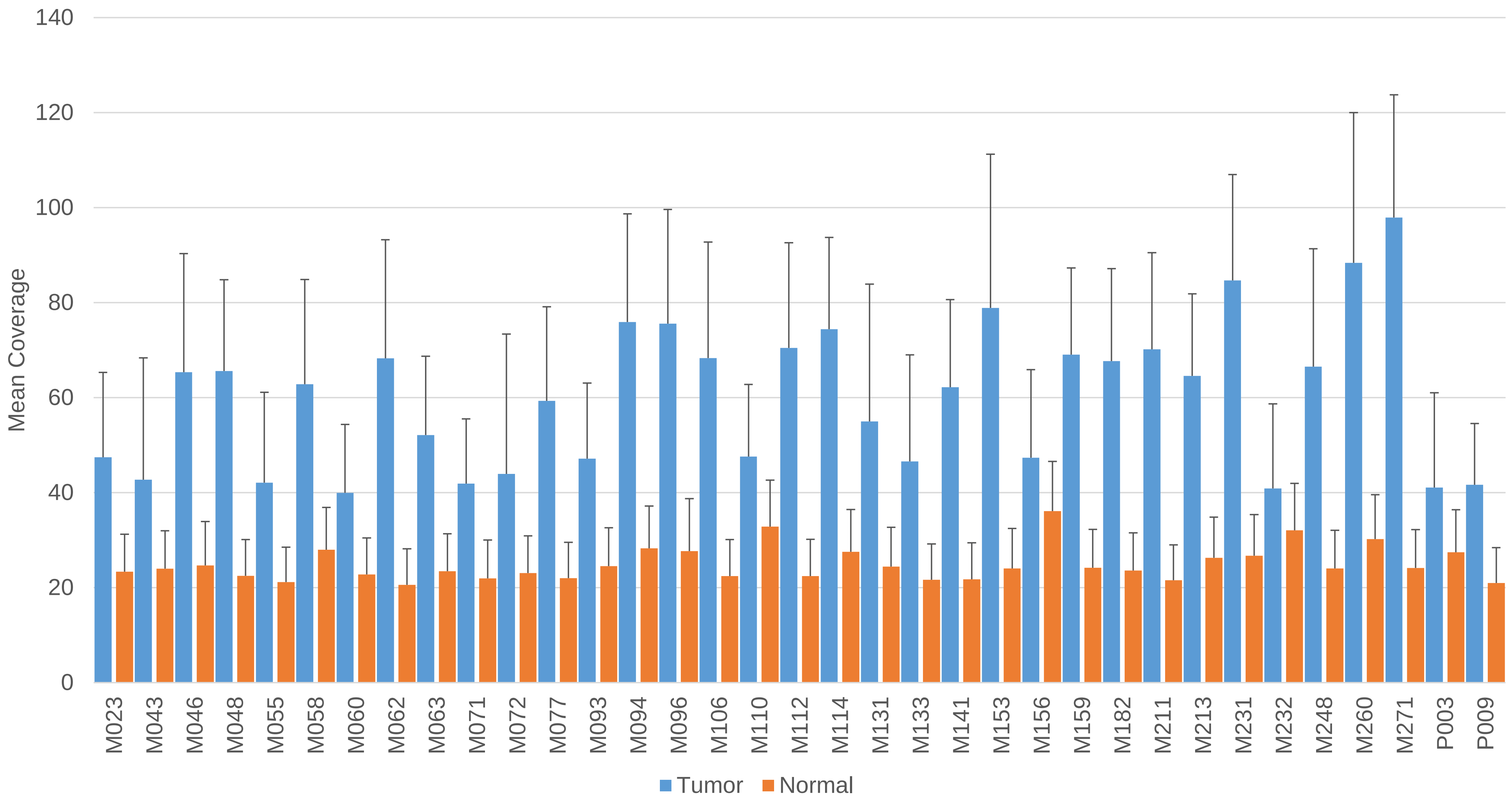

Mean Coverage
0
20
40
60
80
100
120
140
M023
M043
M046
M048
M055
M058
M060
M062
M063
M071
M072
M077
M093
M094
M096
M106
M110
M112
M114
M131
M133
M141
M153
M156
M159
M182
M211
M213
M231
M232
M248
M260
M271
P003
P009
Tumor
Normal

Figure S3

Sample	RECAP	CHORD	B1 & B2-classifier	BRCA status
M248	HRD	HRD	B1 & B2-like	germline BRCA1 mut
M131	HRD	HRD	B1 & B2-like	BRCA1 methylation
M232	HRD	HRD	B1 & B2-like	WT
M182	HRD	HRD	B1-like	BRCA1 methylation
M231	HRD	HRD	B2-like	germline BRCA2 mut
M317	HRD	HRD	NS	germline BRCA2 mut
M365	HRD	HRD	NS	BRCA1 methylation
M260	HRD	HRP	B1 & B2-like	WT
M106	HRD	HRP	B1 & B2-like	BRCA2 VUS
M077	HRD	HRP	B1-like	WT
M362	HRD	NS	B1-like	germline BRCA2 mut
M094	HRD	HRP	B2-like	WT
M096	HRD	HRP	Spor-like	somatic BRCA2 mut
M271	HRD	HRP	Spor-like	WT
M156	HRD	HRP	Spor-like	WT
M211	HRD	HRP	Spor-like	BRCA2 VUS
M093	HRD	HRP	Spor-like	WT
M282	HRD	HRP	NS	WT
M390	HRD	HRP	NS	WT
P002	HRD	NS	B1 & B2-like	germline BRCA2 mut
M188	HRD	NS	B1 & B2-like	germline BRCA2 mut
M270	HRD	NS	B1-like	WT
M057	HRD	NS	B1-like	somatic BRCA2 mut
M275	HRD	NS	B2-like	germline BRCA2 mut
M119	HRD	NS	Spor-like	BRCA1 methylation
M277	HRD	NS	Spor-like	BRCA1 methylation
M141	HRi	HRD	Spor-like	BRCA1 methylation
M367	HRi	HRD	NS	WT
M055	HRi	HRP	B2-like	WT
M313	HRi	HRP	B1-like	WT
M298	HRi	HRP	B1-like	BRCA2 VUS
M253	HRi	NS	B1-like	WT
M278	HRi	NS	B2-like	WT
M112	HRi	HRP	Spor-like	WT
M209	HRi	NS	Spor-like	WT
M273	HRP	HRD	NS	mono-allelic germline BRCA2 mut
M062	HRP	HRP	B1 & B2-like	WT
M046	HRP	HRP	B2-like	WT
M072	HRP	HRP	B2-like	BRCA2 VUS
M213	HRP	HRP	B2-like	WT
P003	HRP	HRP	B2-like	WT
M023	HRP	HRP	Spor-like	WT
M043	HRP	HRP	Spor-like	BRCA1 VUS
M048	HRP	HRP	Spor-like	WT
M058	HRP	HRP	Spor-like	WT
M060	HRP	HRP	Spor-like	WT
M063	HRP	HRP	Spor-like	WT
M071	HRP	HRP	Spor-like	WT
M110	HRP	HRP	Spor-like	WT
M114	HRP	HRP	Spor-like	WT
M133	HRP	HRP	Spor-like	WT
M153	HRP	HRP	Spor-like	WT
M159	HRP	HRP	Spor-like	WT
P009	HRP	HRP	Spor-like	BRCA2 VUS
M252	HRP	HRP	NS	WT
M314	HRP	HRP	NS	WT
M294	HRP	HRP	NS	WT
M299	HRP	HRP	NS	WT
M336	HRP	HRP	NS	WT
M359	HRP	HRP	NS	WT
M363	HRP	HRP	Spor-like	WT
M368	HRP	HRP	NS	WT
M392	HRP	HRP	NS	WT
M396	HRP	HRP	NS	WT
M100	HRP	NS	B1-like	WT
M109	HRP	NS	B2-like	WT
M018	HRP	NS	Spor-like	WT
M021	HRP	NS	Spor-like	WT
M061	HRP	NS	Spor-like	WT
M074	HRP	NS	Spor-like	WT
M181	HRP	NS	Spor-like	WT

Figure S4

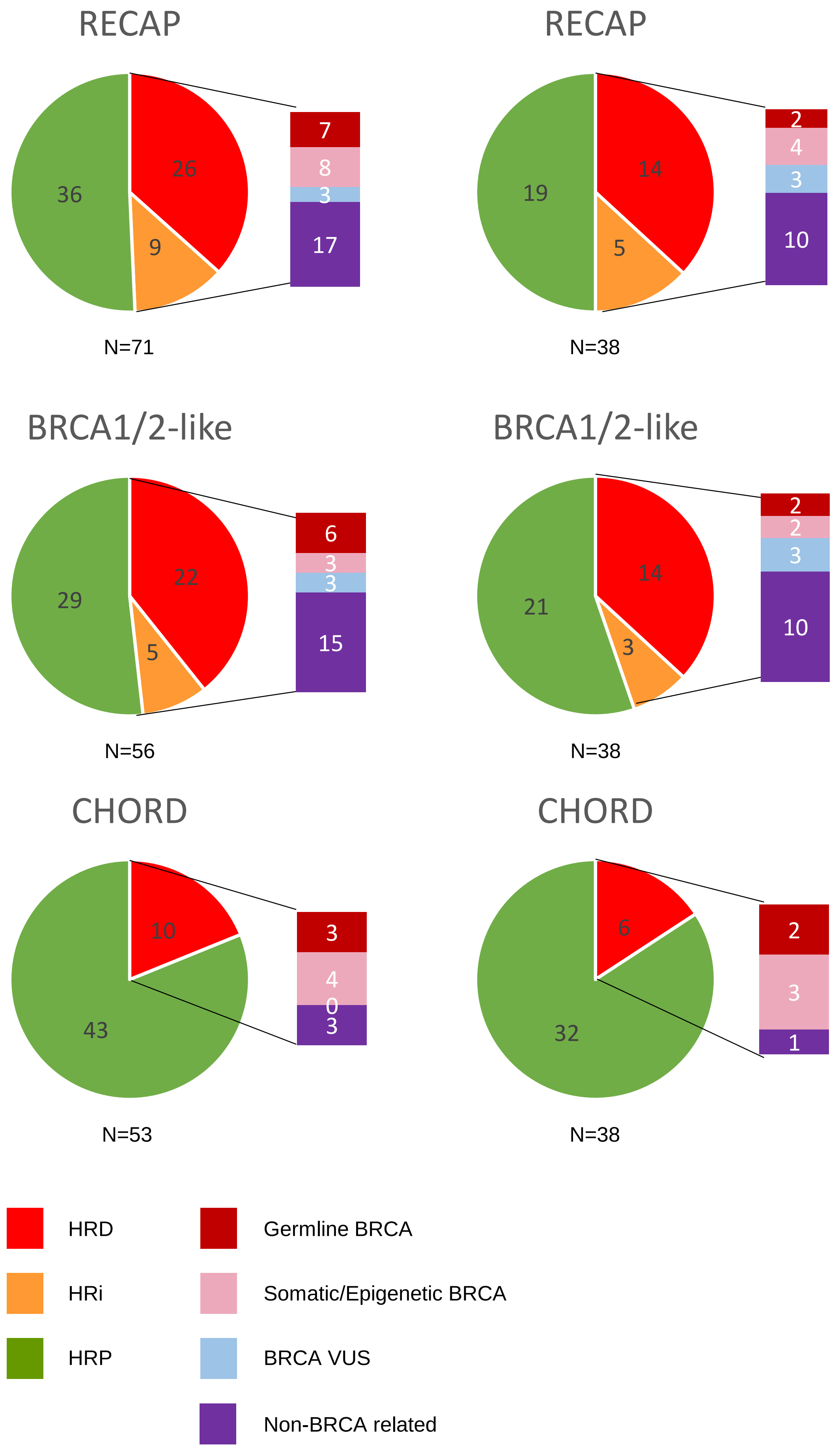

RECAP
36
26
9
7
8
3
17
N=71
RECAP
19
14
5
2
4
3
10
N=38
BRCA1/2-like
29
22
5
6
3
3
15
N=56
BRCA1/2-like
21
14
3
2
2
3
10
N=38
CHORD
10
43
3
4
0
3
N=53
CHORD
6
32
2
3
1
N=38
HRD
HRi
HRP
Germline BRCA
Somatic/Epigenetic BRCA
BRCA VUS
Non-BRCA related

Figure S5

Tumor	HRDetect score	HRDetect	CHORD score	CHORD
**M023**	0,024	proficient	0	proficient
**M043**	0,094	proficient	0	proficient
**M046**	0,034	proficient	0.002	proficient
**M048**	0,002	proficient	0.002	proficient
**M055**	0,289	proficient	0	proficient
**M058**	0,000	proficient	0	proficient
**M060**	0,001	proficient	0	proficient
**M062**	0,213	proficient	0	proficient
**M063**	0,001	proficient	0	proficient
**M071**	0,002	proficient	0	proficient
**M072**	0,000	proficient	0	proficient
**M077**	0,026	proficient	0.004	proficient
**M093**	0,001	proficient	0	proficient
**M094**	0,019	proficient	0	proficient
**M106**	0,157	proficient	0	proficient
**M110**	0,000	proficient	0	proficient
**M112**	0,002	proficient	0.002	proficient
**M114**	0,000	proficient	0.034	proficient
**M131**	1,000	deficient	0.94	deficient
**M141**	0,982	deficient	0.912	deficient
**M153**	0,000	proficient	0	proficient
**M156**	0,002	proficient	0	proficient
**M159**	0,000	proficient	0.008	proficient
**M182**	0,998	deficient	0.91	deficient
**M211**	0,188	proficient	0	proficient
**M213**	0,030	proficient	0	proficient
**M231**	0,967	deficient	0.864	deficient
**M232**	0,995	deficient	0.618	deficient
**M248**	0,998	deficient	0.904	deficient
**M260**	0,032	proficient	0	proficient
**M271**	0,000	proficient	0.002	proficient
**P003**	0,001	proficient	0	proficient
**P009**	0,003	proficient	0	proficient

Figure S6

Comparison BRCA1/2-like classifier and CHORD

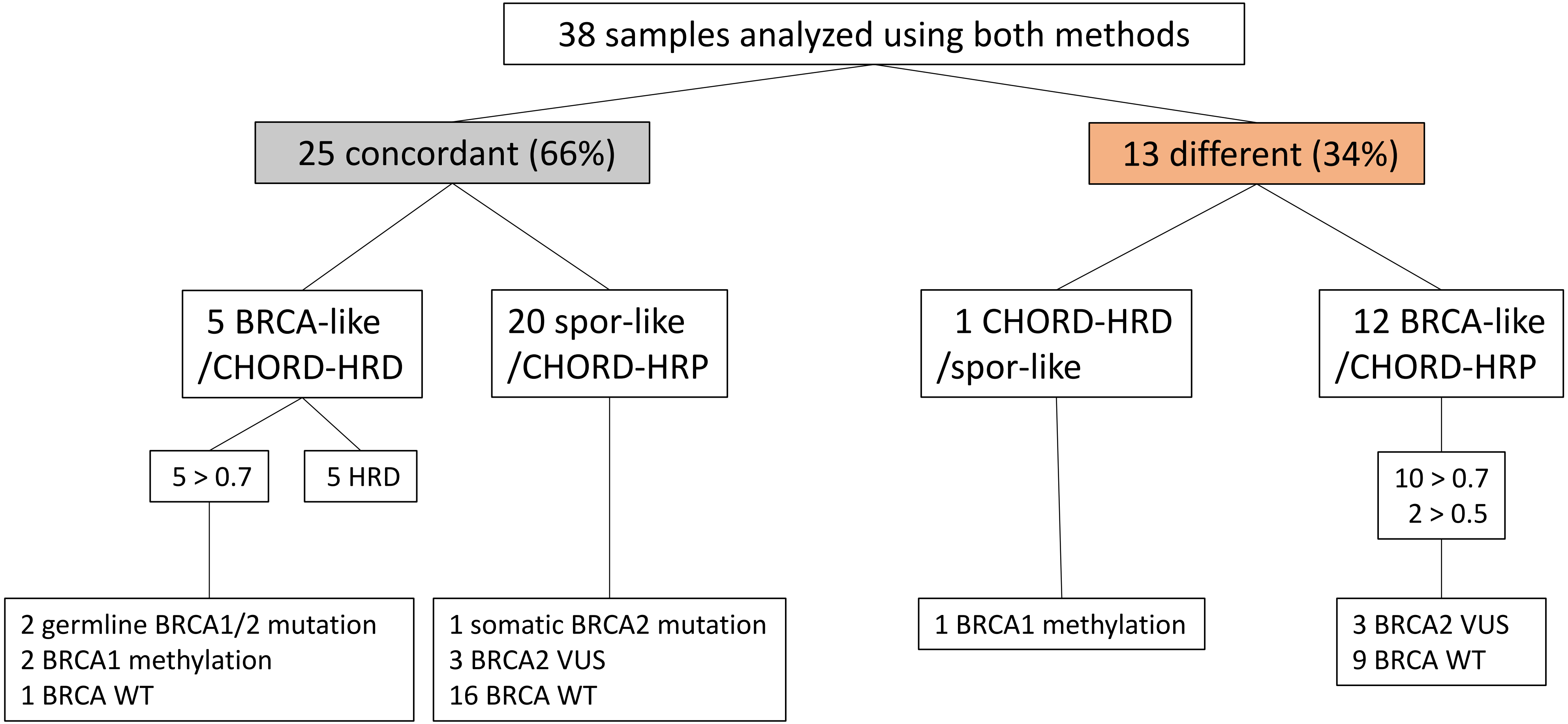

Figure S7

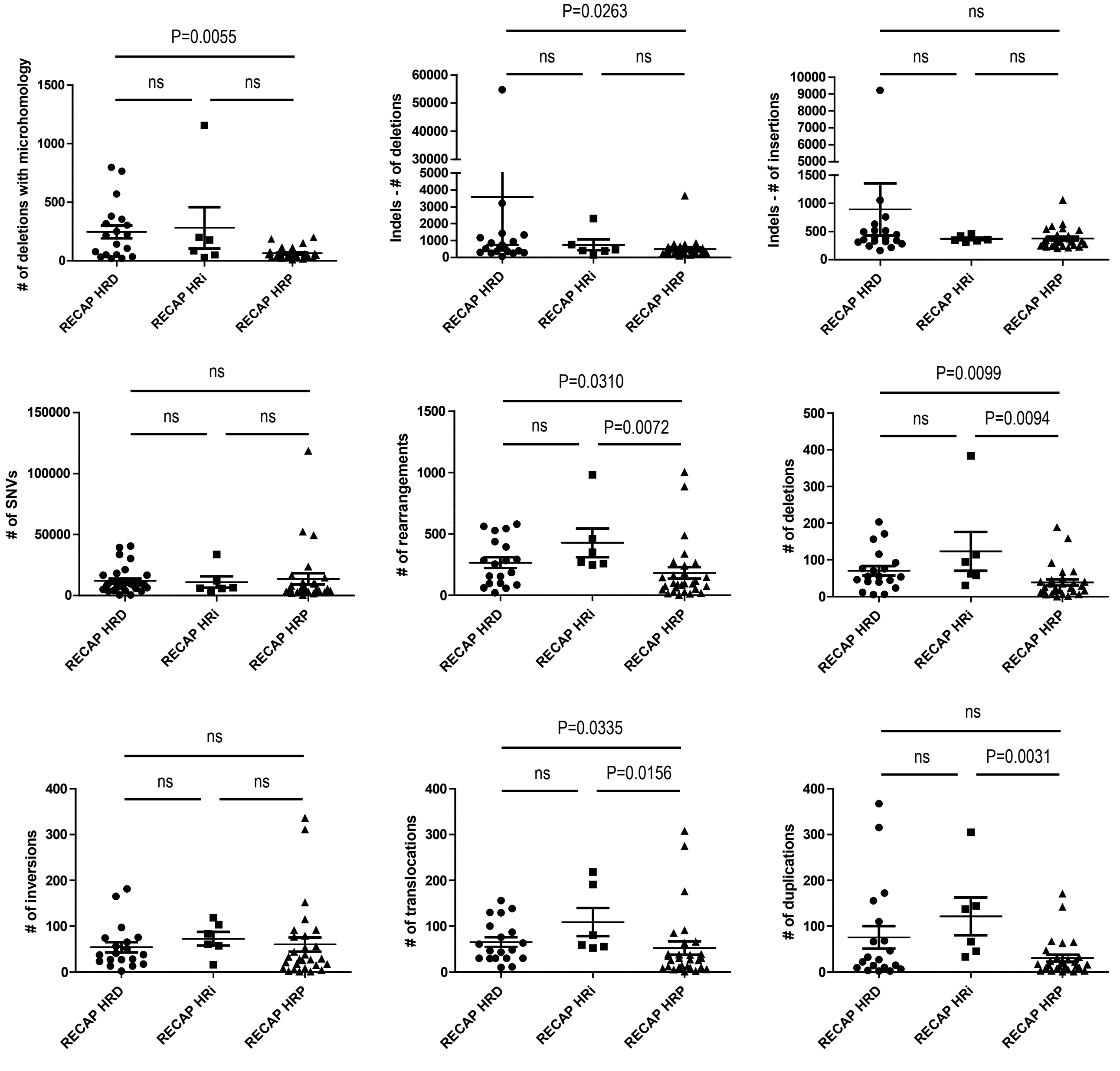
P=0.0055
ns
ns
# of deletions with microhomology
1500
1000
500
0
RECAP HRD
RECAP HRi
RECAP HRP
P=0.0263
ns
ns
Indels - # of deletions
60000
50000
40000
30000
5000
4000
3000
2000
1000
0
RECAP HRD
RECAP HRi
RECAP HRP
ns
ns
ns
Indels - # of insertions
10000
9000
8000
7000
6000
5000
1500
1000
500
0
RECAP HRD
RECAP HRi
RECAP HRP
ns
ns
ns
# of SNVs
150000
100000
50000
0
RECAP HRD
RECAP HRi
RECAP HRP
P=0.0310
ns
P=0.0072
# of rearrangements
1500
1000
500
0
RECAP HRD
RECAP HRi
RECAP HRP
P=0.0099
ns
P=0.0094
# of deletions
500
400
300
200
100
0
RECAP HRD
RECAP HRi
RECAP HRP
ns
ns
ns
# of inversions
400
300
200
100
0
RECAP HRD
RECAP HRi
RECAP HRP
P=0.0335
ns
P=0.0156
# of translocations
400
300
200
100
0
RECAP HRD
RECAP HRi
RECAP HRP
ns
ns
P=0.0031
# of duplications
400
300
200
100
0
RECAP HRD
RECAP HRi
RECAP HRP

Figure S8

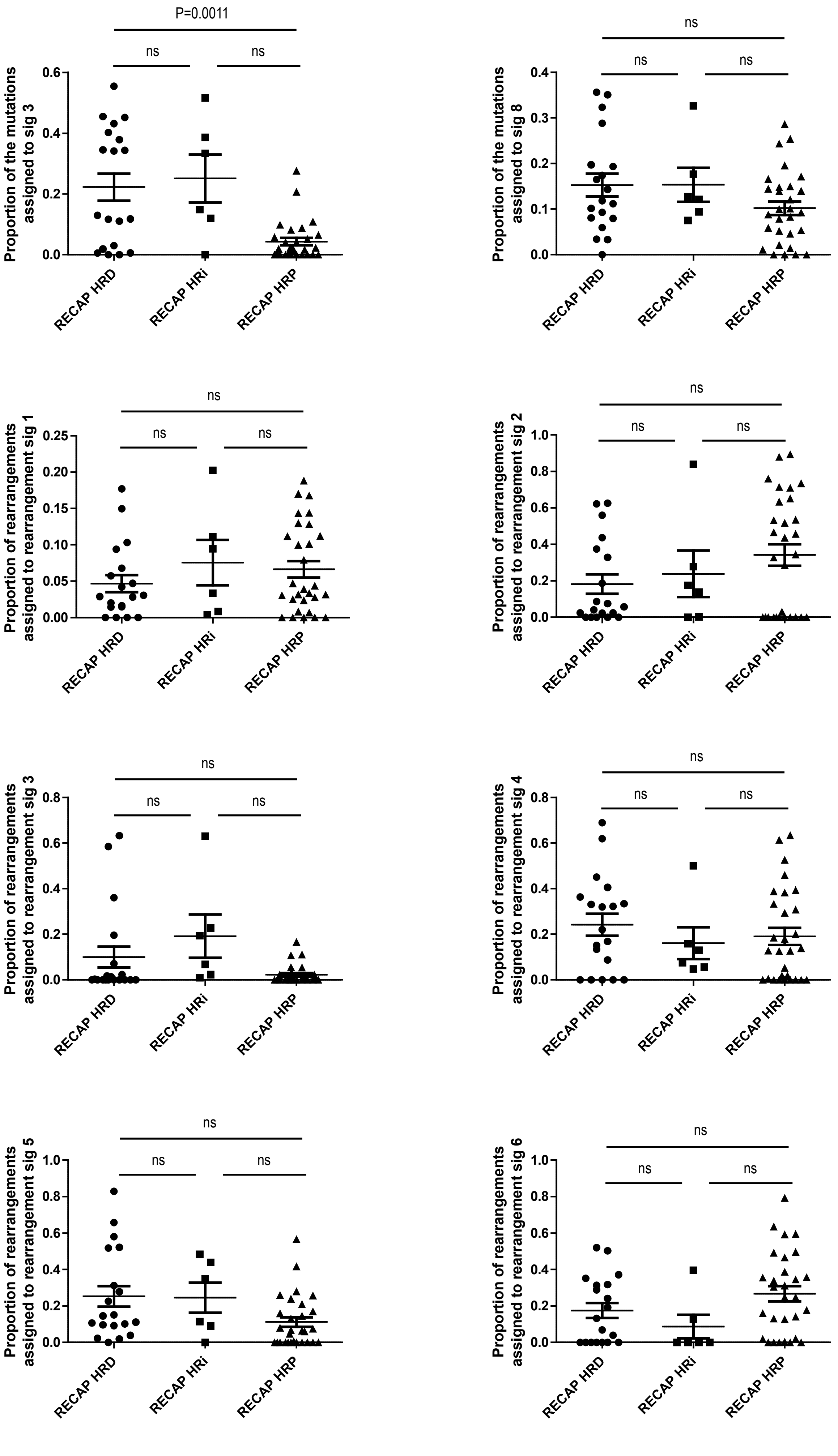
P=0.0011
ns
ns
Proportion of the mutations assigned to sig 3
0.6
0.4
0.2
0.0
RECAP HRD
RECAP HRi
RECAP HRP
ns
ns
ns
Proportion of the mutations assigned to sig 8
0.4
0.3
0.2
0.1
0.0
RECAP HRD
RECAP HRi
RECAP HRP
ns
ns
ns
Proportion of rearrangements assigned to rearrangement sig 1
0.25
0.20
0.15
0.10
0.05
0.00
RECAP HRD
RECAP HRi
RECAP HRP
ns
ns
ns
Proportion of rearrangements assigned to rearrangement sig 2
1.0
0.8
0.6
0.4
0.2
0.0
RECAP HRD
RECAP HRi
RECAP HRP
ns
ns
ns
Proportion of rearrangements assigned to rearrangement sig 3
0.8
0.6
0.4
0.2
0.0
RECAP HRD
RECAP HRi
RECAP HRP
ns
ns
ns
Proportion of rearrangements assigned to rearrangement sig 4
0.8
0.6
0.4
0.2
0.0
RECAP HRD
RECAP HRi
RECAP HRP
ns
ns
ns
Proportion of rearrangements assigned to rearrangement sig 5
1.0
0.8
0.6
0.4
0.2
0.0
RECAP HRD
RECAP HRi
RECAP HRP
ns
ns
ns
Proportion of rearrangements assigned to rearrangement sig 6
1.0
0.8
0.6
0.4
0.2
0.0
RECAP HRD
RECAP HRi
RECAP HRP

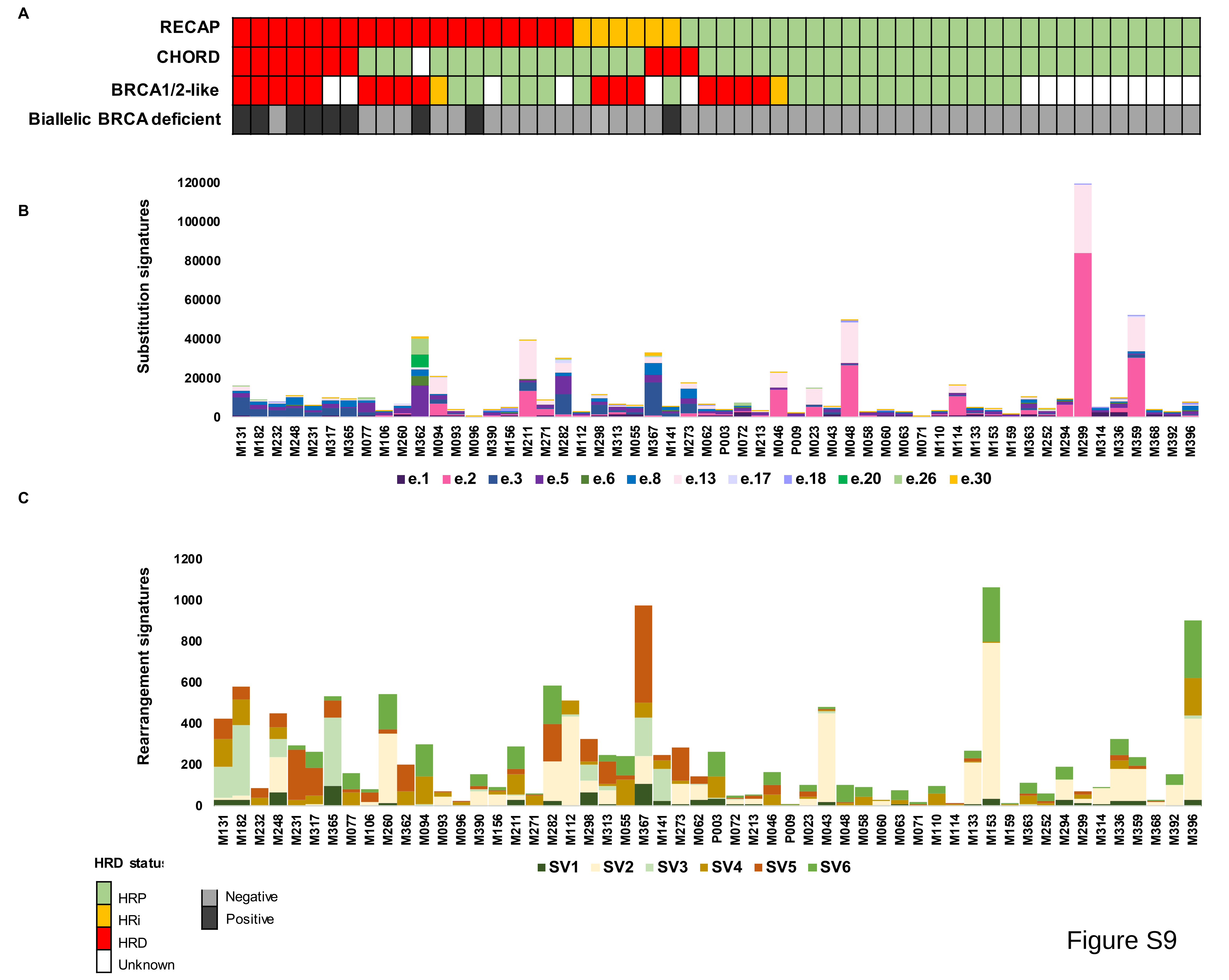

A
RECAP
CHORD
BRCA1/2-like
Biallelic BRCA deficient
B
Substitution signatures
120000
100000
80000
60000
40000
20000
0
M131 M182 M232 M248 M231 M317 M365 M077 M106 M260 M362 M094 M093 M096 M390 M156 M211 M271 M282 M112 M298 M313 M055 M367 M141 M273 M062 P003 M072 M213 M046 P009 M023 M043 M048 M058 M060 M063 M071 M110 M114 M133 M153 M159 M363 M252 M294 M299 M314 M336 M359 M368 M392 M396
e.1 e.2 e.3 e.5 e.6 e.8 e.13 e.17 e.18 e.20 e.26 e.30
C
Rearrangement signatures
1200
1000
800
600
400
200
0
M131 M182 M232 M248 M231 M317 M365 M077 M106 M260 M362 M094 M093 M096 M390 M156 M211 M271 M282 M112 M298 M313 M055 M367 M141 M273 M062 P003 M072 M213 M046 P009 M023 M043 M048 M058 M060 M063 M071 M110 M114 M133 M153 M159 M363 M252 M294 M299 M314 M336 M359 M368 M392 M396
SV1 SV2 SV3 SV4 SV5 SV6
HRD status
HRP
HRi
HRD
Unknown
Negative
Positive

Figure S9

Figure S10

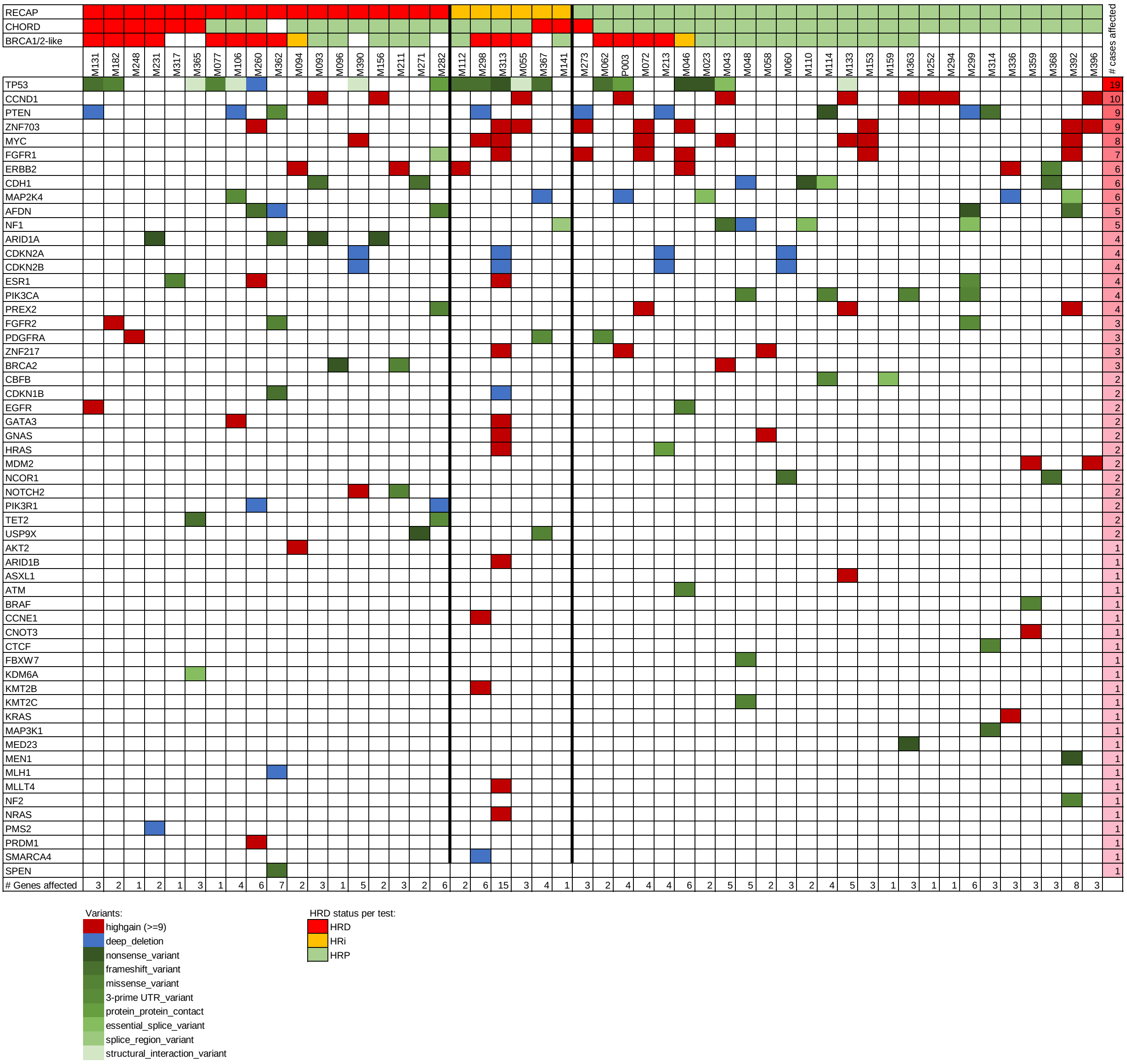
RECAP
CHORD
BRCA1/2-like
M131 M182 M248 M231 M317 M365 M077 M106 M260 M362 M094 M093 M096 M390 M156 M211 M271 M282 M112 M298 M313 M055 M367 M141 M273 M062 P003 M072 M213 M046 M023 M043 M048 M058 M060 M110 M114 M133 M153 M159 M363 M252 M294 M299 M314 M336 M359 M368 M392 M396
cases affected
TP53 19
CCND1 10
PTEN 9
ZNF703 9
MYC 8
FGFR1 7
ERBB2 6
CDH1 6
MAP2K4 6
AFDN 5
NF1 5
ARID1A 4
CDKN2A 4
CDKN2B 4
ESR1 4
PIK3CA 4
PREX2 4
FGFR2 3
PDGFRA 3
ZNF217 3
BRCA2 3
CBFB 2
CDKN1B 2
EGFR 2
GATA3 2
GNAS 2
HRAS 2
MDM2 2
NCOR1 2
NOTCH2 2
PIK3R1 2
TET2 2
USP9X 2
AKT2 1
ARID1B 1
ASXL1 1
ATM 1
BRAF 1
CCNE1 1
CNOT3 1
CTCF 1
FBXW7 1
KDM6A 1
KMT2B 1
KMT2C 1
KRAS 1
MAP3K1 1
MED23 1
MEN1 1
MLH1 1
MLLT4 1
NF2 1
NRAS 1
PMS2 1
PRDM1 1
SMARCA4 1
SPEN 1
Genes affected 3 2 1 2 1 3 1 4 6 7 2 3 1 5 2 3 2 6 2 6 15 3 4 1 3 2 4 4 4 6 2 5 5 2 3 2 4 5 3 1 3 1 1 6 3 3 3 3 8 3
Variants:
highgain (>=9)
deep_deletion
nonsense_variant
frameshift_variant
missense_variant
3-prime UTR_variant
protein_protein_contact
essential_splice_variant
splice_region_variant
structural_interaction_variant
HRD status per test:
HRD
HRi
HRP

Figure S11

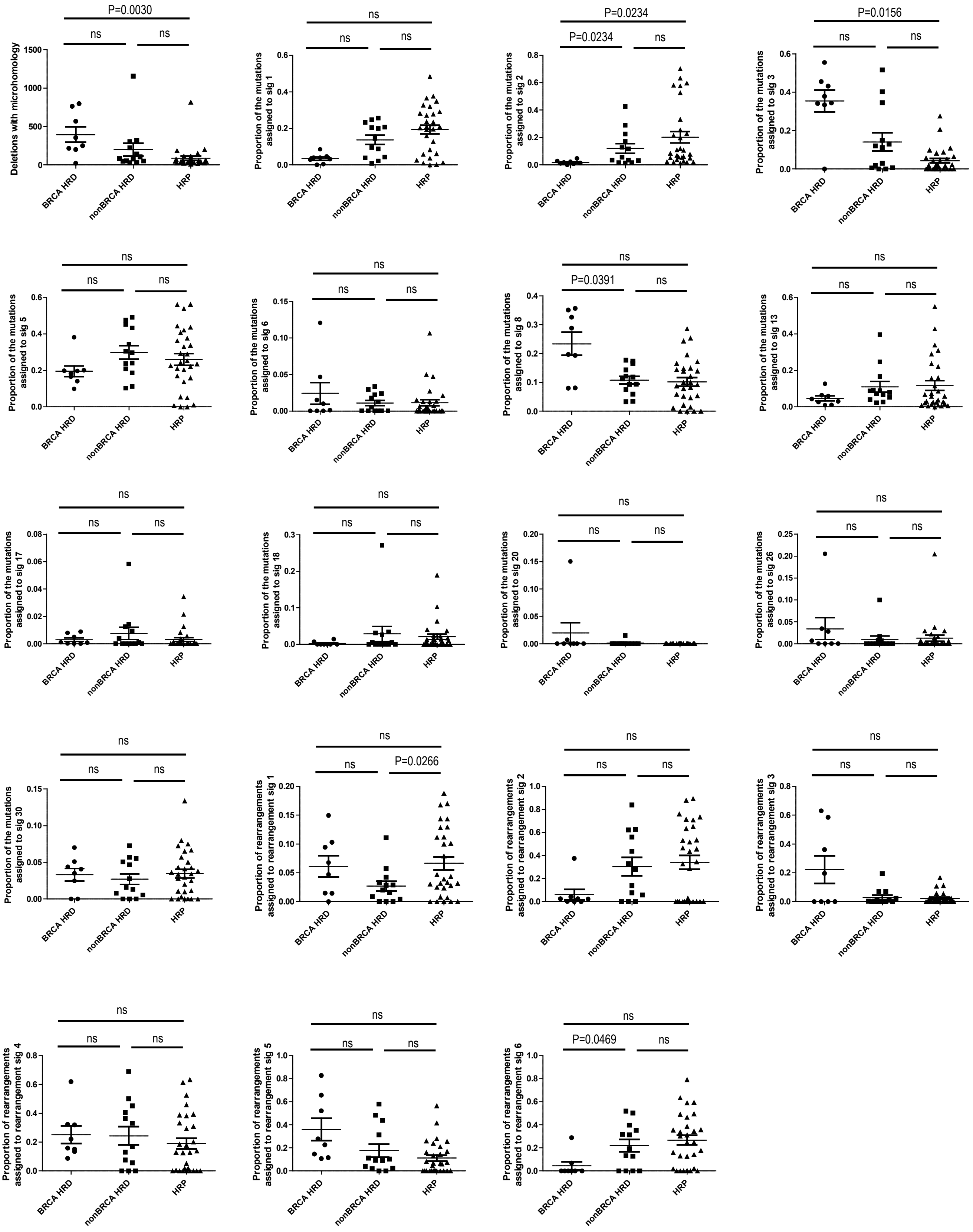

P=0.0030
ns
ns
Deletions with microhomology
BRCA HRD
nonBRCA HRD
HRP
Proportion of the mutations assigned to sig 1
Proportion of the mutations assigned to sig 2
P=0.0234
P=0.0234
Proportion of the mutations assigned to sig 3
P=0.0156
Proportion of the mutations assigned to sig 5
Proportion of the mutations assigned to sig 6
Proportion of the mutations assigned to sig 8
P=0.0391
Proportion of the mutations assigned to sig 13
Proportion of the mutations assigned to sig 17
Proportion of the mutations assigned to sig 18
Proportion of the mutations assigned to sig 20
Proportion of the mutations assigned to sig 26
Proportion of the mutations assigned to sig 30
Proportion of rearrangements assigned to rearrangement sig 1
P=0.0266
Proportion of rearrangements assigned to rearrangement sig 2
Proportion of rearrangements assigned to rearrangement sig 3
Proportion of rearrangements assigned to rearrangement sig 4
Proportion of rearrangements assigned to rearrangement sig 5
Proportion of rearrangements assigned to rearrangement sig 6
P=0.0469
