# Supplementary material for: Functional RECAP (REpair CAPacity) assay identifies homologous recombination deficiency undetected by DNA-based BRCAness tests
Source: Oncogene. 2022 Jun 3;41(26):3498–506. doi: 10.1038/s41388-022-02363-1 (PMC9232391; doi:10.1038/s41388-022-02363-1)
Supplement: Supplementary file 4 — Supplementary figure legends [file 41388_2022_2363_MOESM4_ESM.docx]

**Supplementary Figure Legends**

**Table S1. Clinical data for the 71 breast cancer cases.**

**Table S2. WGS data and CHORD scores for 54 breast cancer cases.**

**Figure S1: Example of RAD51 immunofluorescence read-out.** Immunofluorescent images showing RAD51 foci formation in a control patient, whereas a patient with a *BRCA* mutation shows absence of RAD51 foci. Blue = DAPI, green = Geminin, red = RAD51.

**Figure S2: WGS coverage for each paired tumor and germline sample.**

**Figure S3: HRD classification of 71 breast cancer cases according to three different HRD tests.** Red color indicates HRD, orange HRi and green HRP. NS: no score available. WT: wild-type.

**Figure S4: Distribution of HRD among the different selections of tumors**. Numbers of tumors that were found HRP/HRi/HRD by the different HRD tests in the largest selection of tumors for that specific test (left pie chart) and in the selection of tumors that were subjected to all three HRD tests (right pie chart; n=38). The cause for HRD/HRi (germline BRCA, somatic/epigenetic BRCA, BRCA VUS, non-BRCA related) is depicted in the bar graphs.

**Figure S5: Comparison of whole genome sequencing based HRD prediction methods: CHORD and HRDetect.** For both methods tumors with scores of >0.7 are HRD, scores of <0.7 are HRP.

**Figure S6: Comparison of BRCA1/2-like classifier and CHORD algorithm.** Scores are as in Figure 2.

**Figure S7: Comparison of somatic mutational landscape in RECAP-HRD, RECAP-HRi and RECAP-HRP tumors.** Graphs illustrate difference in SNVs, indels and structural rearrangements between HRD and HRP tumors for the RECAP test. Mann-Whitney U-test was used in the comparisons; p-values are shown; ns = p-values > 0.05.

**Figure S8: Comparison of mutational signatures in RECAP-HRD, RECAP-HRi and RECAP-HRP tumors.** Comparison of proportions of substitution signatures previously reported to be associated with HRD and six rearrangement signatures. Graphs illustrate difference in relative contributions of signatures between HRD and HRP tumors for the RECAP test. Wilcoxon signed rank test was used in the comparisons; p-values are shown; ns = p-values > 0.05.

**Figure S9: Absolute contribution of somatic mutational signatures in breast cancers enriched for HRD**. A:. HRD status per HRD test (CHORD, RECAP or BRCA1/2-like classifier) and biallelic *BRCA1/2* inactivation is depicted for each sample. B: The absolute numbers of twelve substitution signatures and C: rearrangement signatures per tumor.

**Figure S10: Breast cancer driver genes affected in the HRD enriched breast cancer cohort.** Previously, 93 breast cancer driver genes were identified through analysis of a large cohort (n=1332) of sporadic breast cancer genomes [34]. Fifty-seven out of these 93 previously identified BC driver genes contained a somatic mutation, deep deletion or high gain in at least one tumor. The table shows all somatic mutations, deep deletions and high gains (≥9) in the 93 BC driver genes for 50 samples. There were no somatic events in the selected genes in four samples (P009, M063, M071 and M232). Type of somatic mutation is shown (green color scale). HRD status for the three HRD tests is shown.

**Figure S11: Comparison of somatic landscape in biallelic BRCA deficient, non-BRCA related RECAP-HRD and RECAP-HRP tumors.** Comparison of the number of deletions with microhomology, proportions of 12 substitution signatures and six rearrangement signatures. For these comparisons, HRD tumors with BRCA VUSes were excluded, since it remains uncertain whether these tumors are BRCA related or non-BRCA related. Graphs illustrate difference in relative contributions of signatures between biallelic BRCA deficient, non-BRCA related HRD and HRP tumors for the RECAP test. Mann-Whitney U-test was used in the comparison of deletions with microhomology and wilcoxon signed rank test was used in the comparisons of relative contribution of signatures; p-values are shown; ns = p-values > 0.05.
